# microRNA production in *Arabidopsis*

**DOI:** 10.3389/fpls.2023.1096772

**Published:** 2023-01-19

**Authors:** Ning Ding, Bailong Zhang

**Affiliations:** Ministry of Education Key Laboratory of Cell Activities and Stress Adaptations, School of Life Sciences, Lanzhou University, Lanzhou, China

**Keywords:** miRNA biogenesis, DCL, AGO, microprocessors, Arabidopsis

## Abstract

In plants, microRNAs (miRNAs) associate with ARGONAUTE (AGO) proteins and act as sequence-specific repressors of target gene expression, at the post-transcriptional level through target transcript cleavage and/or translational inhibition. MiRNAs are mainly transcribed by DNA-dependent RNA polymerase II (POL II) and processed by DICER LIKE1 (DCL1) complex into 21∼22 nucleotide (nt) long. Although the main molecular framework of miRNA biogenesis and modes of action have been established, there are still new requirements continually emerging in the recent years. The studies on the involvement factors in miRNA biogenesis indicate that miRNA biogenesis is not accomplished separately step by step, but is closely linked and dynamically regulated with each other. In this article, we will summarize the current knowledge on miRNA biogenesis, including *MIR* gene transcription, primary miRNA (pri-miRNA) processing, miRNA AGO1 loading and nuclear export; and miRNA metabolism including methylation, uridylation and turnover. We will describe how miRNAs are produced and how the different steps are regulated. We hope to raise awareness that the linkage between different steps and the subcellular regulation are becoming important for the understanding of plant miRNA biogenesis and modes of action.

## 1 Introduction

**Figure 1:**
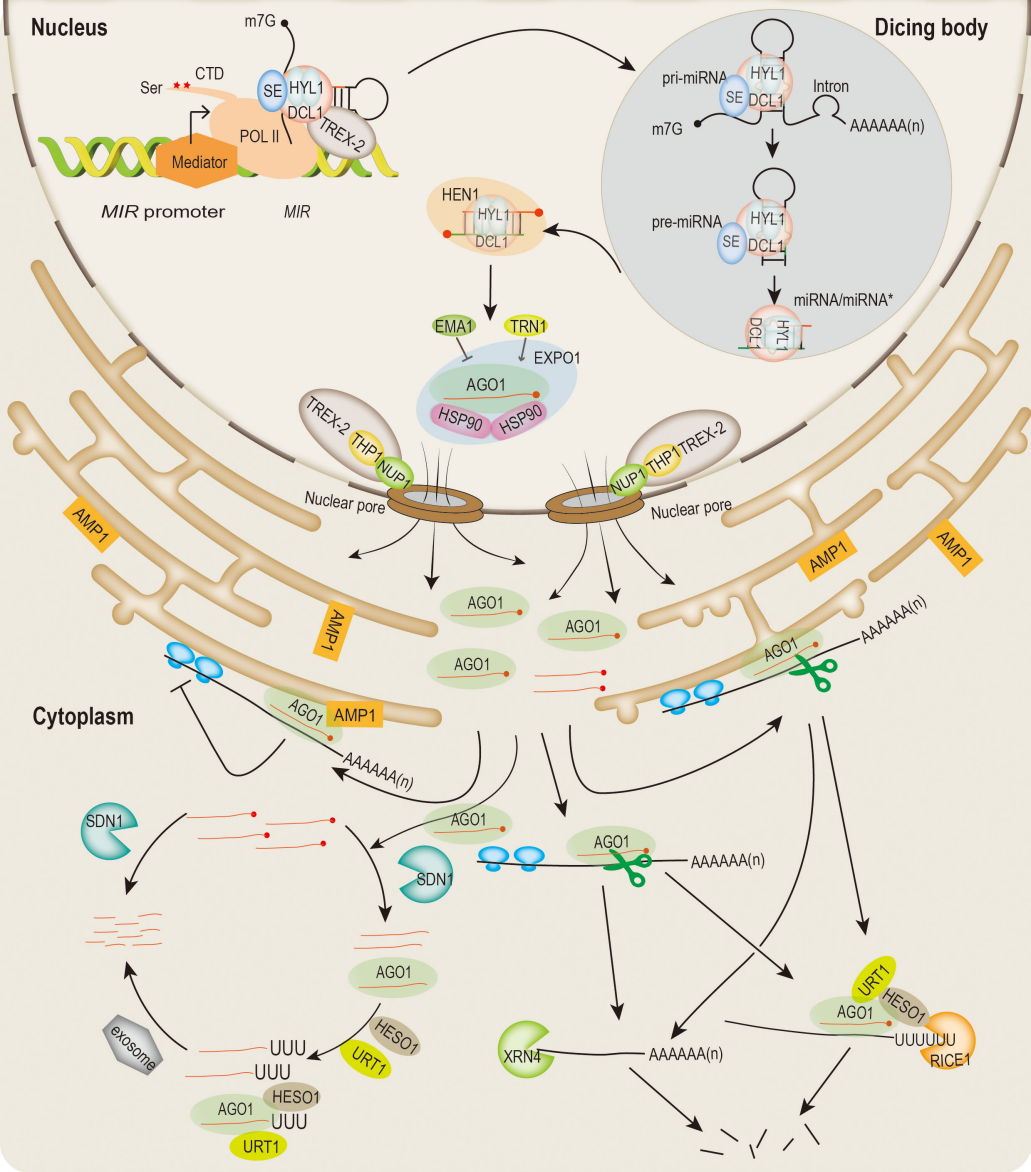
Overview of miRNA biogenesis, miRISC assembling and export, and modes of action in plants. *MIR* genes are transcribed by POL II and regulated by multiple cotranscriptional regulators (such as mediator, DCL1 complex, TREX-2, ect.). The C-terminus of POL II can be phosphorylated by kinases during the transcription process. Pri-miRNA is processed at the dicing bodies (DCL1, HYL1 and SE). Pre-miRNA is subsequently processed by DCL1 into an imperfect miRNA/miRNA* duplexes. HEN1 methylates the 3’ end of the miRNA/miRNA*. Mature miRNAs are loaded into AGO1 protein in the nucleus. MiRISCs are exported into the cytoplasm through a CRM1 (EXPO1)/NES-dependent manner *via* the TREX-2/NUP1 facilitation pathway. EMA1 and TRN1 can negatively and positively regulate miRNA loading, respectively. Translocation of miRNAs from the nucleus to the cytoplasm directs target transcript cleavage and translation repression. AGO1 mediates cleavage of miRNA targets sequences, and followed by degradation of cleaved fragments. Translation repression occurs on membrane-bound polysomes (MBPs), and requires endoplasmic reticulum (ER)-localized AMP1. Degradation of miRNA begins with the removal of the methyl group at the 3’ end by the SDN1 family of 3’-5’ exonucleases, followed at 3’ end uridylation by the nucleotidyl transferases HESO1 and/or URT1. In addition, SDN1 and HESO1/URT1 can act on both AGO-bound miRNAs and free miRNAs in the cytoplasm. Free miRNAs are also degraded by SDN1 directly. For the target mRNAs cleaved by miRISC, RICE1 is responsible for degrading the uridylated target mRNA 5’ fragment. The 5’-3’ exonuclease XRN4 can degrade the 3’ fragment of target RNA.

Plant miRNAs are a class of 21∼22 nucleotides (nt) endogenous small RNAs that repress gene expression either through target RNA cleavage and/or translational inhibition (Achkar et al., 2016; Yu et al., 2017b). In plants, miRNAs are involved in various cellular and physiology processes and impact plant development, growth, and stress responses (D'Ario et al., 2017; Yu et al., 2019). In the past years, the roles of miRNAs in many biological processes have been gradually revealed. In the meantime, researchers have also established the molecular framework of miRNA biogenesis and modes of action in plants (**Figure 1**).

Plant *MIRNA* (*MIR*) genes are transcribed by RNA POL II rendering 5’ capped (m7Gppp) and 3’ polyadenylated primary transcripts (pri-miRNAs) (Xie et al., 2005; Knop et al., 2017). The transcription of *MIR* genes is similar to the POL II-mediated eukaryotic genes, with their own promoter region, and their expression is regulated by a variety of transcriptional processes (Zhang et al., 2015). Pri-miRNA is a long stem-loop structure with an imperfectly matched in the middle of the stem that is considered undergo two sequential reactions to form miRNA/miRNA* duplexes in highly specialized nuclear dicing bodies (D-bodies) (Fang and Spector, 2007). In the first step, pri-mRNA is processed into a short precursor (pre-mRNA) by the microprocessors with central components of DCL1, HYPONASTIC LEAVES 1 (HYL1, also known as DRB1), and SERRATE (SE) (Rogers and Chen, 2013; Achkar et al., 2016). Pre-miRNA is further processed by DCL1 into a double-stranded miRNA duplex (Yu et al., 2017b; Li and Yu, 2021a). The nascent miRNA/miRNA* duplexes usually contain 2 nt overhang and two hydroxyl groups at both 3’ ends. The 2’ OH of the 3’ terminal nucleotide is immediately methylated by HUA ENHANCER 1 (HEN1), and the guide strand (miRNA) in the double-stranded RNA is loaded into AGO1 or AGO1 paralogs to form the miRNA-induced silencing complex (miRISC), while the passenger strand (miRNA*) is degraded (Yu et al., 2005; Singh et al., 2018). The assembly process of miRISC mainly occurs in the nucleus, which is attributed to the nucleo-cytosolic shuttling protein AGO1 (Bologna et al., 2018). Furthermore, nuclear plant miRNAs-AGO1 are exported to the cytoplasm through the CRM1 (EXPO1)/NES-dependent manner *via* TREX-2 and nucleoporin protein (NUP1) facilitated pathway (Bologna et al., 2018; Zhang et al., 2020). It has been shown that the conformation of the AGO1 is modified to accommodate the process of RISC assembly, which also needs to be facilitated by many cofactors such as HEAT-SHOCK PROTEIN 90 (HSP90) and CYCLOPHILIN 40 (CYP40) (Iki et al., 2010; Iwasaki et al., 2010; Iki et al., 2012; Nakanishi, 2016). In addition, ENHANCED MIRNA ACTIVITY1 (EMA1) and TRANSPORTIN1 (TRN1) are members of the AGO1-interacting importin-β family of proteins, which negatively and positively regulate miRNA loading into AGO1, respectively (Wang et al., 2011; Cui et al., 2016).

The actions of cytoplasmic miRNAs occur mainly through the repression of their target mRNAs in two ways: transcripts cleavage and translational repression. miRISC-catalyzed mRNA cleavage is accomplished by the PIWI domain of AGO1 or other paralogs proteins. After cleavage, the 3’ fragments are subsequently degraded by 5’-3’ exonuclease EXORIBONUCLEASE 4 (XRN4) (Souret et al., 2004). The 5’ fragments are uridylated by HEN1 SUPPRESSOR 1 (HESO1) and UTP: RNA URIDYLYLTRANSFERASE 1 (URT1) at the 3’ end both *in vivo* and *in vitro* (Ren et al., 2014; Wang et al., 2015). In addition, RISC-INTERACTING CLEARING 3’-5’ EXORIBONUCLEASE 1 (RICE1) are involved in degradation of uridylated 5’ fragments (Zhang et al., 2017b). Moreover, the cytoplasmic exosome consists of subunits such as SUPERKILLER 2 (SKI2), SKI3 and SKI8, which are responsible for degrading the 5’ fragment generated by RISC (Branscheid et al., 2015). Translational repression also plays a vital role in miRNA-mediated post-transcriptional regulation. In fact, as translation repression was observed less frequently than transcript cleavage, the mechanism of translation repression still needs to be elucidated. Nonetheless, multiple factors are identified to participate in miRNA-mediated translational repression (Brodersen et al., 2008; Yang et al., 2012; Li et al., 2013; Reis et al., 2015). Notably, ALTERED MERISTEM PROGRAM1 (AMP1), an integral membrane protein associated with AGO1 on the endoplasmic reticulum (ER), which is responsible for miRNA-directed translational repression at the protein level (Li et al., 2013).

To maintain miRNA homeostasis, the abundance of miRNAs needs to be precisely regulated. The miRNA/miRNA* is stabilized by HEN1 through 3’-terminal 2’-O-methylation (Chen et al., 2002). HESO1 and URT1 can uridylate the 3’ unmethylated miRNA, leading to miRNA degradation (Ren et al., 2014; Wang et al., 2015). Free miRNAs can be degraded by SMALL RNA DEGRADING NUCLEASE 1 (SDN1) (Ramachandran and Chen, 2008). For miRNAs bound to AGO1, removal of methyl group at the 3’ end is also initially mediated by SDN1 (Ramachandran and Chen, 2008). Subsequently, a few of proteins are responsible for the degradation of miRNAs at different stages (Ramachandran and Chen, 2008; Ren et al., 2012a; Zhao et al., 2012b; Tu et al., 2015).

This review summarizes our current understanding of the *MIR* gene transcription, pri-miRNA cotranscriptional processing, pri-miRNA modification, miRNA AGO1 loading and nuclear export, turnover and degradation of miRNAs. We aim to provide an updated view of miRNA biogenesis pathways in plants.

## 2 Regulation of pri-miRNA accumulation

### 2.1 *MIR* gene transcription

**Figure 2:**
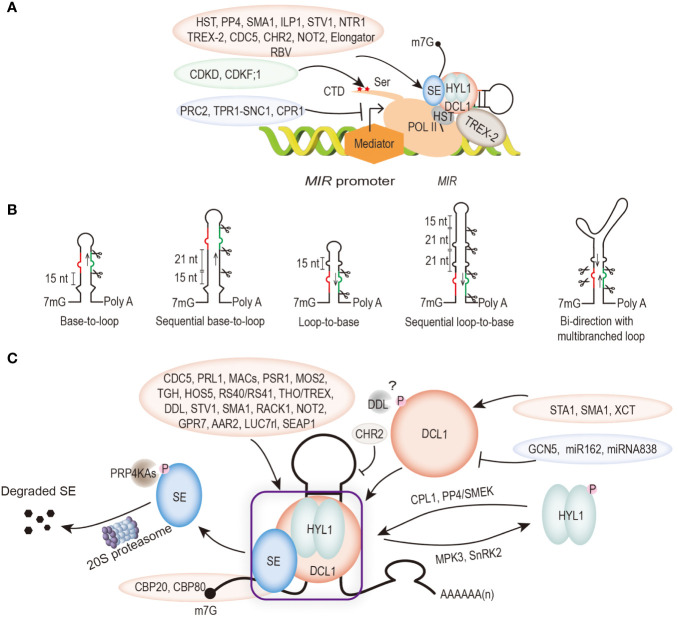
Diagram of the principal steps in microRNA (miRNA) biogenesis. Positive and negative modulators are marked with light pink and light purple ellipses, respectively. **(A)** Regulation of *MIR* transcription. Multiple transcription regulators are required to regulate POL II-mediated *MIR* transcription. The activity of POL II is also regulated by phosphorylation of its C-terminal structural domain (CTD). **(B)** Structures of pri-miRNAs affect their processing patterns. DCL1 recognizes a small bulge near the base of the stem-loop secondary structure of pri-miRNA. Base-to-loop or sequential base-to-loop processing of pri-miRNAs. Some pri-miRNAs are cut first at ∼15 nt distal to the loop to release miRNA/miRNA* duplex, but some pri-miRNAs with long stems are initially cut at 15 nt distal to the loop, and each subsequent cut is ∼21 nt to release miRNA/miRNA*. Loop-to-base or sequential loop-to-base processing pattern of pri-miRNAs. DCL1 recognizes a small loop at the end of the stem, and cut at 15 nt toward the base of the stem. Some pri-miRNAs with long stem loops are processed through multiple cleavages from the proximal to the loop at 21 nt intervals. Bidirectional processing of pri-miRNAs with a multibranched terminal loop. **(C)** Regulation of pri-miRNA processing. Processing pri-miRNAs require the use of core dicing bodies (DCL1, SE, and HYL1) as well as several other accessory components. The abundance and activity of DCL1, SE, and HYL1 are regulated by several protein factors and miRNAs. Core microprocessor of phosphorylation status affects the efficiency of pri-miRNA processing. Phosphorylation of SE mediated by PRP4KAs and followed be degraded by the 20S proteasome. CBP80 and CBP20 are subunits of the nucleocapsid binding complex (CBC), which interact with SE. They bind to the m^7^G cap structure of the 5’ end pri-mRNA to prevent degradation. The phosphorylation of DCL1 is mediated by DAWDLE (DDL) to affect pri-miRNA processing. The dephosphorylated and phosphorylated forms of HYL1 are mediated by a series of phosphatases (CPL1, PP4/SMEK) and kinases (MPK3 and SnRK2). In addition, the microprocessor can be synergized by many proteins to promote pri-miRNA processing (marked with light pink ellipse), while CHR2 inhibits its processing.

The length of *Arabidopsis MIR* genes ranges from 319 bp (*MIR165a*) to 4975 bp (*MIR472*) (Stepien et al., 2017). The transcription process also requires the recruitment of mediator complexes and phosphorylation of the C-terminal domain (CTD) of the largest subunit of POL II, which together mediate the early transcriptional events of pri-mRNAs (Kim et al., 2011; Achkar et al., 2016). Unlike siRNAs, transcription and processing of POL II-mediated *MIR* genes are regulated by transcription regulators that act as positive and/or negative roles (**Figure 2A**). These regulators include CYCLIN-DEPENDENT KINASE F;1 (CDKF1) and CYCLIN-DEPENDENT KINASE Ds (CDKDs) phosphatases that catalyze the Ser phosphorylation of the CTD, and collaborate with transcriptional activators such as elongator complex, Negative on TATA less2 protein (NOT2), cell division cycle 5 (CDC5), SHORT VALVE 1 (STV1), and repressors such as F-Box protein CONSTITUTIVE EXPRESSER OF PR GENE 1 (CPR1), disease resistance protein CONSTITUTIVE 1 (SNC1) and its interacting protein TOPLESS RELATED 1 (TPR1) (Hajheidari et al., 2012; Wang et al., 2013; Zhang et al., 2013; Fang et al., 2015a; Li et al., 2017; Su et al., 2017; Cai et al., 2018). Moreover, REDUCTION IN BLEACHED VEIN AREA (RBV) with a WD40 repeats domain, can facilitate the transcription of *MIR* into pri-miRNAs by enhancing the occupancy of POL II at the *MIR* promoter to promote miRNA production (Liang et al., 2022). What have to be remarked is the vital effects of chromatin remodeling factors and histone epistasis modifications in the *MIR* genes transcription process. For instance, general control non-repressed protein5 (GCN5) targets a subset of *MIR* genes that are deposited by H3K14 to promote its miRNA accumulation (Kim et al., 2009). However, polycomb repressive complex 2 (PRC2) represses transcription by increasing the H3K27me3 methylation level of the *MIR156A/C* promoter (Xu et al., 2016).

Noteworthy, increasing evidence suggested that the transcription process of *MIR* genes is accompanied by pri-miRNAs processing (**Figure 2A**) (Kim et al., 2011; Wang et al., 2013; Fang et al., 2015a; Cambiagno et al., 2021). The microprocessor component DCL1 can be recruited to the *MIR* loci depending on its association with the POL II-accessory complex mediator and elongator (Kim et al., 2011; Wang et al., 2013; Fang et al., 2015a). It was shown that Protein phosphatase 4 (PP4) promotes HYL1 to associate with *MIR* promoters Wang SK et al., 2019. In addition, PRE-MRNA PROCESSING PROTEIN 40 (PRP40), the U1 snRNP auxiliary protein, positively regulates the recruitment of SE to *MIR* genes (Stepien et al., 2022). Moreover, it was shown that *Arabidopsis* TREX-2 complex promotes *MIR* gene transcription, pri-miRNA processing and miRISC nuclear export (Zhang et al., 2020). In the TREX-2 defective mutant *thp1*, DCL1 and HYL1 *MIR* gene recruitment ability was impaired, indicating that processing of pri-miRNA may linked with *MIR* gene transcription and miRISC export (Zhang et al., 2020). *Arabidopsis* HASTY (HST), the ortholog of mammalian EXPORTIN5, interacts with mediator subunit MED37 and DCL1 to form miRNA biogenesis complex at *MIR* gene loci, promoting the transcription and processing of pri-miRNA (Cambiagno et al., 2021). It was recently found that transcription of the plant R-loops near the transcription start site (TSS) region of the *MIR* gene is coupled with miRNA processing, as proven by the plant native elongating transcripts sequencing (plaNET-seq) method (Gonzalo et al., 2022). These observations indicate that the linkages between microprocessor, POL II and export factors are widespread in plant, suggesting that processing of plant miRNAs are coupled with *MIR* transcription and mature miRNA export.

### 2.2 Pri-miRNA stability regulation

The transcribed *MIR* genes are mostly processed by the microprocessor, while the rest unprocessed pri-miRNAs trigger RNA degradation by 5’-3’ exonucleases (XRNs) and 3’-5’ exonuclease complex exonucleases to maintain the amounts of pri-miRNAs (Chekanova et al., 2007; Gy et al., 2007; Kurihara et al., 2012). Inhibition of XRN2 and XRN3 by 3’-phosphadenosine 5’-phosphate (PAP) leads to increased accumulation of both pri-miRNA and mature miRNAs (Fang et al., 2019). In addition, RNA helicase HEN2 acts as a cofactor of the nucleoplasmic exosome and is responsible for the degradation of a few pri-miRNAs (Lange et al., 2011). Another novel zinc finger protein, SOP1, co-localizes with HEN2 in nucleoplasmic foci and participates in the degradation of a subset of nucleoplasmic exosome substrates (Hématy et al., 2016).

It was also shown that the components of miRNA biogenesis participate in pri-miRNA degradation and stabilization. Besides its function in pri-miRNA processing, SE can interact with the nuclear exosome targeting (NEXT) complex to degrade pri-miRNAs (Bajczyk et al., 2020). Conversely, *hen2* and *sop1* mutants enhance the stability and accumulation of some pri-miRNAs in *hyl1* mutant, but no similar effects were found in *dcl1* mutant, suggesting that HYL1 also plays a noncanonical role in protecting pri-miRNAs from nuclear exosome attack (Gao et al., 2020). Moreover, MAC5, a component of the MOS4-associated complex, interacts with SE and the stem loop of pri-miRNAs to facilitate pri-miRNA processing and protect pri-miRNAs from SE-dependent 5’-3’ exoribonuclease degradation (Li SJ et al., 2020).

There are several other proteins have been shown to protect pri-miRNAs from degradation in unknown pathways. DAWDLE (DDL), as an RNA-binding protein, interacts with both DCL1 and pri-miRNAs to stabilize pri-miRNAs (Yu et al., 2008). *ddl* mutant shows reduced accumulation of both pri-miRNAs and mature miRNAs, but without affecting the *MIR* gene transcription (Yu et al., 2008). In addition, PROTEIN PLEIOTROPIC REGULATORY LOCUS 1 (PRL1) and MAC3 (a U-box type E3 ubiquitin ligase) also have function in stabilizing pri-miRNAs (Zhang et al., 2014; Li et al., 2018a).

## 3 Regulation of miRNA biogenesis by pri-miRNA structure and modification

As the substrates of the microprocessor, pri-miRNAs themselves also affect the processing efficiency in various ways (Li and Yu, 2021a). Unlike in metazoans, plant pri-miRNAs have highly variable sizes and secondary structures. Pri-miRNA secondary structure is one of the important factors which affecting the cleavage site selection and processing efficiency of DCL1 (**Figure 2B**). DCL1 functions as molecular ruler that measures and cleaves pri-miRNAs into 21 nt long mature miRNA duplexes (Macrae et al., 2006). DCL1 processes pri-miRNAs in a base-to-loop manner in two steps (Kurihara and Watanabe, 2004). The first cut is located 15 to 17 nt away from the base of the stem or a bulge or unstructured region within the loop-distal stem. The resulting precursor-miRNA (pre-miRNA) is further cleaved by DCL1 to produce a 21 nt miRNA/miRNA* duplex (Song et al., 2010; Liu et al., 2012). However, some pri-miRNAs with longer hairpins are processed in a base-to-loop manner. The first cut is ∼21 nt proximal to the miRNA/miRNA*, which is likely determined by the presence of a 15 bp stem below the cut site and additional cuts to generate miRNA/miRNA* duplex (Bologna et al., 2013; Zhang et al., 2015). In contrast, some pri-miRNAs such as pri-miR159a and pri-miR319a possess long upper stem and are cleaved by DCL1, resulting in loop-to-base processing mechanism (Bologna et al., 2009). Interestingly, a few pri-miRNAs contain multibranched terminal loops that can be processed bidirectionally, but the base-to-loop processing seem to be more effective, as in the case of pri-miR166C (Zhu et al., 2013). In addition, several young miRNAs (miR822 and miR839) from long inverted repeat (IR) locus are processed by DCL4 instead of DCL1 (Rajagopalan et al., 2006).

Unlike DNA modifications that can dynamically modulate gene expression, some factors in mRNA modifications are critical to the biogenesis of miRNAs (Marquardt et al., 2022). It is known that N6-methyladenosine (m^6^A) modification occurs in plant pri-miRNAs, which can be introduced by adenosine methylesterase (MTA), a homolog of METTL3 (Bhat et al., 2020). MTA can interact with known miRNA biogenesis regulators, such as POL II and Tough (TGH), to promote miRNA biogenesis (Ren et al., 2012b; Bhat et al., 2020). The abundances of m6A and miRNAs are apparently reduced in the *mta* mutant (Bhat et al., 2020). The low level of m^6^A may affect the association between the stem-loop structure of pri-miRNAs and HYL1 (Bhat et al., 2020). Additionally, m^6^A can modulate splicing process through YTH Domain-Containing 1 (YTHDC1) and other several pre-mRNA splicing factors (Xiao et al., 2016). These studies indicate that RNA modifications can occur cotranscriptionally affecting downstream processing events (Marquardt et al., 2022).

## 4 Key roles of microprocessors in the biosynthesis of miRNAs

Extensive studies were published to describe how the nascent pri-miRNAs are processed into mature miRNAs. In plants, DCL1 is the riboendonuclease to process the long pri-miRNAs into mature miRNAs (Macrae et al., 2006). The miRNA dicing complex is composed of DCL1, HYL1 and SE. In the past decades, many factors that directly or indirectly modulate microprocessor activity have been identified (**Figure 2C**) (Li and Yu, 2021a; Zhang et al., 2022). These factors include CDC5, PRL1, Phytophthora Suppressor of RNA Silencing 1 (PSR1), MODIFIER OF SNC1, 2 (MOS2), MAC3, MAC5, MAC7, CAP-BINDING PROTEIN 20 (CBP20) and CBP80, homolog of the DEAD-box pre-mRNA splicing factor Prp28 (SMA1), the pre-mRNA processing factor 6 homolog STABILIZED1 (STA1), HIGH OSMOTIC STRESS GENE EXPRESSION 5 (HOS5), ARGININE/SERINERICH SPLICING FACTOR 40 (RS40) and RS41, the U1 snRNP Subunit LETHAL UNLESS CBC 7 RL (LUC7rl), the THO2 in the THO/TREX complex, GLYCINERICHRBP 7 (GPR7), SE-Associated Protein 1 (SEAP1) etc. also interact with the microprocessor and positively regulate pri-miRNA processing (Laubinger et al., 2008; Ben Chaabane et al., 2013; Wu et al., 2013; Zhang et al., 2013; Koster et al., 2014; Zhang et al., 2014; Chen et al., 2015; Francisco-Mangilet et al., 2015; Qiao et al., 2015; Jia et al., 2017; Knop et al., 2017; Li et al., 2018a; Li et al., 2018b; Li SJ et al., 2020; Li et al., 2021b).

### 4.1 DCL1

Microprocessors are the key plant miRNA biosynthesis machines involved in accurate production of mature miRNAs. The main component in microprocessors is DCL1, which contains two ribonuclease III domains and is responsible for the process from pri-miRNAs to mature miRNAs (Macrae et al., 2006). The null *dcl1* mutants are embryo-lethal, and weak mutants show severe developmental defects such as reduced fertility, late flowering and the typical pleiotropic developmental defects (Schauer et al., 2002). Some of the mutations affect the accumulation of mature miRNAs, while other weak mutations may affect DCL1 own subcellular localization and/or interactions with other proteins, yet they show WT phenotype but lose the accuracy of DCL1 cleavage (Schauer et al., 2002; Stepien et al., 2017).

It has been reported that the transcription level of *DCL1* is fine-tuned by miRNAs (**Figure 2C**). *DCL1* mRNA remains low due to the cleavage of miR162 produced by DCL1 protein, and the transcription process of *DCL1* is aborted with miR838 harbored in exon 14/15 of *DCL1* (Xie et al., 2003; Rajagopalan et al., 2006). Conversely, XAP5 CIRCADIAN TIMEKEEPER (XCT), STA1 and SMA1 promote the transcription of *DCL1 via* direct physical interactions (Ben Chaabane et al., 2013; Fang et al., 2015b; Li et al., 2018b). It has also been reported that the phosphothreonine recognition cleft of the DDL FHA-domain can be bound by the DCL1 fragment, suggesting a phosphorylation-regulated function of DCL1 (Machida and Yuan, 2013). Additional factors include TGH, DDL and MOS2, also interact with DCL1 to enhance its activity (Yu et al., 2008; Ren et al., 2012a; Wu et al., 2013).

### 4.2 HYL1

There are five double-stranded RNA binding (*DRB*) genes in *Arabidopsis* genome, of which HYL1 is one of the essential components of the microprocessor involved in miRNA biosynthesis (Hiraguri et al., 2005). HYL1 can form a homodimer that bind to the stem region of pri-miRNA for its proper processing (Yang et al., 2010; Yang et al., 2014). All members of the DRB family contain two double-stranded RNA binding domains (dsRBDs). One of the dsRBDs in HYL1 can bind to the stem of pri-miRNA and the other can interact with the dsRBD of DCL1 and SE (Hiraguri et al., 2005; Machida et al., 2011). It was reported that two dsRBDs of DCL1 (DCL1-D1/D2) and only the second dsRBD (DCL1-D2) could complement the *hyl1* functions to some extent (Wu et al., 2007; Liu et al., 2013). Loss of function *hyl1-2* mutant is not lethal but displays severe developmental defects such as dwarf growth, reduced root growth rate and altered responses to several hormones (Lu and Fedoroff, 2000). Besides HYL1, DRB2, DRB3 and DRB5 seem to participate in the regulation of gene silencing in a dynamic synergistic or antagonistic manner, while DRB4 is shown to facilitate the production of tasiRNAs and young miRNAs by interacting with DCL4 (Achkar et al., 2016).

HYL1 can interact with DCL1 to facilitate efficient and precise pri-miRNA processing (Kurihara et al., 2006). HYL1 also impacts some pri-miRNAs splicing and strand selection from miRNA/miRNA* duplexes in AGO1 loading (Eamens et al., 2009; Szarzynska et al., 2009; Ben Chaabane et al., 2013). In spite of the unclear mechanism of the precise function of HYL1 in the cytoplasm and how it is translocated from the nucleus to the cytoplasm, KETCH1 (karyopherin enabling the transport of the cytoplasmic HYL1 has been reported to be responsible for the import of HYL1 into the nucleus (Zhang et al., 2017a). Besides, the cytoplasmic fraction of HYL1 may also associate with AGO1 to regulate its distribution in polysomes and promote miRNA-mediated mRNA translation repression (Yang et al., 2021).

HYL1 acts as a short-lived phosphoprotein and its activity is precisely regulated by a series of phosphatase and protein kinases (**Figure 2C**). Mitogen-Activated Protein Kinase 3 (MPK3) and SNF1-related protein kinase (SnRK2) alter HYL1 stabilization through phosphorylating S42 and S159 sites (Raghuram et al., 2015; Yan et al., 2017). However, C-Terminal Domain Phosphatase-like 1 (CPL1), PP4 and Suppressor of MEK 1 (SMEK1) play roles in the dephosphorylation of HYL1 protein and promote miRNA biogenesis (Manavella et al., 2012; Su et al., 2017). AAR2, a homolog of U5 snRNP in yeast and humans, plays a role in splicing and miRNA biogenesis in plants, and promotes HYL1 dephosphorylation to produce the active form HYL1 (Fan et al., 2022). So far, all the available evidence suggest that the non-phosphorylated version of HYL1 as the active form (Mencia et al., 2022).

### 4.3 SE

The third core microprocessor component is the SE protein, which contains a core region of an N-terminal α-helix, a middle region as well as a C-terminal C_2_H_2_ zinc-finger domain and a partial nuclear localization signal (NLS) (Machida et al., 2011). As an ortholog of the mammalian arsenic resistance protein 2 (Ars2), SE also affects *Arabidopsis* developmental processes such as leaf development, growth rate and inflorescence architecture (Grigg et al., 2005). Similar to *DCL1*, null mutant of *SE* also exhibits embryonic lethality, while other weak mutants such as *se-1* and *se-2* exhibit severe developmental abnormalities with reduced mature miRNA levels (Prigge and Wagner, 2001; Grigg et al., 2005). Earlier reports showed that the core region of SE provides a platform that can directly interact with DCL1 and HYL1 to ensure the three proteins form a complex in the D-bodies (Fang and Spector, 2007; Song et al., 2007). Contrary to this notion, it was recently reported that SE-mediated phase separation is critical for the assembly of D-bodies with diameters of 0.2∼0.8 μm, and the number of such droplets often has 2 to 4 in the nucleus of *Arabidopsis* cells (Xie et al., 2021). Consistent with the functions of DCL1 and HYL1, *se* results in reduced levels of mature miRNAs, increased levels of pri-miRNAs and defects in pri-miRNA splicing (Lobbes et al., 2006).

SE can directly interact with nuclear mRNA cap-binding complex (CBC) components CBP20 and CBP80/ABH1, which also affect miRNA biogenesis and may be involved in the splicing function of pre-mRNA (Laubinger et al., 2008). RECEPTOR FOR ACTIVATEDC KINASE 1 (RACK1) interacts with SE to promotes pri-miRNA processing (Speth et al., 2013). SE can also interact with the SWI2/SNF2 family member CHR2/BRM to remodel pri-miRNA secondary structure and reduce miRNA accumulation by blocking the activity of downstream microprocessors (Wang et al., 2018). In addition, as a scaffold protein, SE is phosphorylated or dephosphorylated like HYL1 and DCL1 to modulate its protein activity. Pre-mRNA processing 4 kinase A (PRP4KA) and its paralogs directly phosphorylate at least five residues of SE protein for the degradation *via* the 20S proteasome to maintain its metabolic homeostasis (**Figure 2C**) (Li YJ et al., 2020; Wang et al., 2022a). However, it is unclear whether there are other kinases that can phosphorylate SE and affect its local folding and aggregation.

## 5 miRNA stabilization, RISC assembly and nuclear export

The 2’-O-methylation modification of mature miRNAs at their 3’ ends is catalyzed by the methyltransferase HEN1, which represents a key step in miRNA stabilization in plants (**Figure 1**). Null *hen1-2* mutant exhibits a series of developmental defects such as dwarfism, late flowering and short siliques, impaired photomorphogenic and skotomorphogenic (Chen et al., 2002; Tsai et al., 2014), and features of reduced miRNA abundance and miRNA size heterogeneity (Li et al., 2005). The crystal structure shows that the double-stranded RNA (dsRNA) of the miRNA duplex produced by DCL1 as a substrate can be preferentially bound by HEN1 (Huang et al., 2009). HEN1 is localized both in the nucleus and cytoplasm (Fang and Spector, 2007), and the place where methylation occurs remains unknown. Moreover, *in vitro* assays demonstrate HEN1 may directly interact with DCL1 and HYL1 (Baranauskė et al., 2015).

After miRNA maturation, nuclear HYL1 may be required for selecting the miRNA strand from the miRNA duplex and interact with other cofactors to load the guide strand into AGO1 (**Figure 1**) (Eamens et al., 2009; Manavella et al., 2012). AGO1 has conserved NLS and NES sequences conferring the ability to shuttle between the cytoplasm and nucleus (Bologna et al., 2018). This challenges the notion that the loading of mature miRNAs into AGO1 in plants occurs primarily in the cytoplasm as in animals. A recent report showed that RBV can promote the loading of miRNAs into AGO1 (Liang et al., 2022). Some of miRNAs, such as miR165, miR166 and miR160, are found to be poorly loaded into AGO1 after being processed in a co-transcriptional manner, implying that miRNAs processing during transcription take a nuclear export route that avoids AGO1 loading, resulting in the cytoplasmic pool of “free” miRNAs that can move from cell to cell (Gonzalo et al., 2022). This could also explain why HST plays a co-transcriptional role in miRNA biogenesis and cell-autonomously promotes cell-to-cell movement (Brioudes et al., 2021; Cambiagno et al., 2021). For miRNAs loaded illegitimately into AGO1, or non-functional AGO1-miRNA pairs and unloaded AGO1, the F-box protein FBW2 assembles an SCF complex to promote AGO1 degradation, and regulation of FBW2 activity also requires the CURLY LEAF (CLF), a subunit of the polycomb repressor complex 2 (PRC2) (Dalmadi et al., 2019; Ré et al., 2020; Hacquard et al., 2022). The export process requires AGO1 to form a complex with chaperone HSP90 during its association with the methylated mature miRNA/miRNA* duplex (Iki et al., 2010). The activity of HSP90’s chaperone may alter the conformation of AGO1 when it binds or separates from it (Rogers and Chen, 2013). However, this process is promoted by Cyclophilin 40/Squint (CYP40/SQN) containing the TPR domain, and is suppressed by Protein Phosphatase 5 (PP5) (Iki et al., 2010; Iwasaki et al., 2010; Iki et al., 2012).

The nuclear envelope (NE) separates nuclear and cytoplasmic to control the macromolecular exchange, and it has a highly organized double membrane that is morphologically continuous with the endoplasmic reticulum (ER) of eukaryotic cells (Nigg, 1989). The nuclear pore complex (NPC), one of the structural components of NE, is composed of multiple copies of nucleoporin proteins (NUPs) (Strambio-De-Castillia et al., 2010). In *Arabidopsis*, The TREX-2 complex includes SAC3A, SAC3B, SAC3C, DSS1-(I), DSS1-(V), CEN1 and CEN2, which are localized in the NPC basket and are essential for transcription and mRNA export (Lu et al., 2010). It has been reported that the nuclear basket protein and TREX-2 complexes are linked by THP1-NUP1 interactions (Lu et al., 2010). NUP1 and THP1 have also been shown to facilitate nuclear export of miRNAs and AGO1 (Zhang et al., 2020). This links miRNA biosynthesis, assembly of miRISC and nuclear export processes through a cascade of dynamic regulation (**Figure 1**).

## 6 Turnover and degradation of miRNAs

The abundance of miRNAs in cells is balanced by the rate of biogenesis and degradation. In addition to miRNA biogenesis, another factor affecting miRNA levels is miRNA degradation which also includes miRNA 3’ uridylation and 3’ truncation (**Figure 1**) (Yu et al., 2017b). As mentioned above, HEN1 mediated miRNA methylation can protect the small RNA duplex from degradation. For those unmethylated small RNAs, the addition of a U-rich tail at 3’ end is governed by the activity of the nucleotidyl transferase HESO1 and URT1, which is considered as a signal for the degradation (Ren et al., 2012a; Zhao et al., 2012b; Tu et al., 2015). HESO1 is in both the nucleus and cytoplasm, and URT1 is predominantly distributed in the cytoplasm. The null *heso1* mutant partially rescues the developmental defects of the *hen1* mutant, coupled with an increase in miRNA accumulation and a reduction in 3’ uridylation (Zhao et al., 2012b). The point mutation of URT1 (*urt1-3*) partially rescues the fertility defect of *hen1 heso1-2* mutant, and the triple mutant of *hen1-2 heso1-2 urt1-3* does not result in the complete absence of miRNA uridylation, presumably in the presence of other uridyltransferases (Wang et al., 2015). In addition, nucleotidyl transferase proteins 4 (NTP4) was recently reported as a close homolog of HESO1 and URT1 to direct the asymmetric uridylation of miRNA/miRNA* (guide/messenger) duplexes to regulate miRNA accumulation (Kong et al., 2021).

Some miRNAs can also be truncated before being uridylated in *hen1* mutant, implying divergent degradation fates (Zhai et al., 2013). Degradation of miRNAs from both directions involves two separated mechanisms. The involvement of 3’ to 5’ exonucleases in the degradation of mature small RNAs is the SDN family, which belongs to the DEDD superfamily (**Figure 1**) (Ramachandran and Chen, 2008; Wang et al., 2018). *In vitro* assays show SDN1 is unable to degrade U-tailed miRNAs, by contrast, several exonucleases in other eukaryotes prefer uridylated RNAs as substrates in mammals and yeast (Ramachandran and Chen, 2008). Additionally, it has been demonstrated that SDN1 only works on short ssRNAs, not small RNA duplexes, pre-miRNAs, or larger RNAs *in vitro* (Ramachandran and Chen, 2008). Analysis of the small RNA-seq data of *hen1* and *hen1 sdn1 sdn2* plants shows the 3’ truncation of some miRNAs is reduced when there is a lack of SDN1 and SDN2, demonstrating the SDNs are responsible for the 3’ truncation of miRNAs (Yu et al., 2017a). It is possible that SDN1 and HESO1/URT1 modulate the miRNAs that are coupled to AGO1 because truncated and uridylated miRNAs are associated with AGO1 *in vivo* (Zhao et al., 2012a).

The 10 AGO proteins are divided into three clades containing the conserved PAZ, MID and PIWI domains (Carbonell, 2017). Of these, AGO1 not only acts as an effector but may also serve as a refuge to protect miRNAs from degradation, since the abundance of many miRNAs is reduced in *ago1* mutants (Vazquez et al., 2004). As a paralog of AGO1, AGO10 plays an opposite role to AGO1 in miRNA stabilization. AGO10 is involved in regulating stem cell and leaf polarity, miR165/166 is at high abundance in *ago10* mutants, while overexpression of *AGO10* reduces the accumulation of miR165/166 (Yu et al., 2017a; Wang JL et al., 2019). AGO10 binds miR165/6 with higher affinity than AGO1 and promotes its degradation, and rather than protecting miR165/6 (Zhu et al., 2011; Zhou et al., 2015). Therefore, different effects on the stability of the miRNAs are determined by which AGO proteins are loaded.

## 7 Conclusions and future perspectives

In order to adapt to environmental variability for survival, sessile organisms have adopted a range of responsive strategies. MiRNAs are essential players in the regulation of proper growth and development by cleaving of target mRNA and translational repression in various manners. Players of the miRNA biogenesis pathway and their activities are central to the generation of miRNA, and they can be mediated by regulators at different tissues and developmental stages. In this review, we focused on the dynamic control of the key steps in miRNA biogenesis pathway and also exhibit a hierarchical model of how these processes are regulated. We also emphasized the importance of how the miRNA processing is linked with *MIR* gene transcription and miRNA export, especially on the subcellular regulation of plant miRNAs. Nevertheless, studies on how and why plant miRNA biogenesis steps have to be tightly linked are still largely unknown. In addition, how the major protein complexes in each step dynamically interact or communicate is still unknow. For example, there is no studies on whether POL II dynamically associate with D-bodies and nuclear pore complex, or whether the miRNA loci have direct interaction with D-bodies. Moreover, how does the nuclear miRNA loading cooperate with miRISC export still remain to be further explored. Furthermore, *Arabidopsis* or other terrestrial or semiterrestrial plants with multi-organizational systems pose research barriers compared to animal cells, so a breakthrough in deep sequencing technology at the single-cell level in plants is still needed. However, in plants, utilizing canonical forward or reverse genetic merits can serve us to screen novel cofactors that regulate miRNA production pathways.

## Author contributions

ND drafted the manuscript and the figures. BZ conceived the idea and revised the manuscript. All authors contributed to the article and approved the submitted version.
